# Right Coronary Artery - Looped, Astray and Muzzled: A case report

**DOI:** 10.1186/1749-8090-10-S1-A27

**Published:** 2015-12-16

**Authors:** Anitha Guru, Naveen Kumar, AM Prasad

**Affiliations:** 1 Department of Anatomy, Melaka Manipal Medical College, Manipal Campus, Manipal University, Manipal, Karnataka, 576104, India

## Background/Introduction

The right coronary artery (RCA) arises from the right aortic sinus. In its course in relation to the atrioventricular groove (AVG), it is submerged in the adipose tissue of the epicardium. It continues towards the posterior interventricular groove often crossing the crux of the heart.

## Aims/Objectives

To trace the RCA and determine the variation in its course

## Method

The heart of a well embalmed male cadaver is dissected to trace the RCA in the right AVG. The branches are traced to determine the variation in the course of the RCA.

## Results

During the exposure of RCA, the right AVG was found unoccupied. Further probing did reveal the atrial branches bearing a proximal origin, but no ventricular branches in the right AVG. On tracing the atrial branches to their origin, the RCA was visible. The RCA had a normal origin from the right aortic sinus. Soon after, instead of being submerged in the adipose tissue, it produced a unique pattern of looping with two corners and the loop rose above the surface of the heart. The space underneath the loop was filled with adipose tissue. The RCA then exited the AVG and went astray into the myocardium of the right ventricle. Within the wall of right ventricle, the artery initially pursued a superficial course giving rise to ventricular branches in the direction of the left ventricle. Few of the ventricular branches produced elevations on the surface of the right ventricle. For the further part, the RCA did not enter the AVG and hence did not reach the crux. Due to its intra-myocardial course, the right marginal artery was not seen on the surface of the heart. The coronary sinus was the sole relation to the AVG.

## Discussion/Conclusion

In this case, the RCA and its ventricular branches appear muzzled by the myocardium of the right ventricle. Such a variation in the course of RCA accompanied by the unique looping pattern is rare. It may pose a tough challenge to the radiologists and cardiologists in performing various procedures such as coronary stenting involving the RCA

